# Walking on Uneven Terrain with Hexapod Robots Having Underactuated Legs and Articulated Body

**DOI:** 10.3390/biomimetics11020132

**Published:** 2026-02-11

**Authors:** Ioan Doroftei

**Affiliations:** 1 Mechanical Engineering, Mechatronics and Robotics Department, “Gheorghe Asachi” Technical University of Iasi, 43 D. Mangeron Blvd., 700050 Iasi, Romania; ioan.doroftei@academic.tuiasi.ro; 2 Technical Sciences Academy of Romania, 26 Dacia Blvd., 030167 Bucharest, Romania; 3 Academy of Romanian Scientists, 3 Ilfov, 050044 Bucharest, Romania

**Keywords:** hexapod walking robot, articulated body, underactuated leg, active compliance, force distribution, rollover recovery

## Abstract

Hexapod walking robots are a subject of intense research in the existing literature. To move effectively in natural terrain, these robots must be able to adapt to surface irregularities. While most existing designs employ sophisticated technical solutions for the leg mechanisms, none of these projects allow for combined roll and pitch movements of the body segments. This paper addresses this gap, presenting the concept of a hexapod robot with a body formed of three segments connected by two active universal joints. This unique architecture allows the robot to locomote on both sides and autonomously recover from a rollover event. The robot’s legs are underactuated, utilizing a passive spring element to simplify the mechanical design and control system while maintaining effective terrain adaptation capabilities. Experimental results are presented and discussed, validating the theoretical model and demonstrating the effectiveness of the proposed solution on varied terrains.

## 1. Introduction

Hexapod walking robots, inspired by biological locomotion, are designed to traverse complex environments. With six legs, a hexapod robot can maintain balance even if one or two legs are lifted, making it more stable than bipeds or quadrupeds, particularly on uneven terrain such as rough, rocky, or soft surfaces where wheeled or tracked robots might get stuck. Engineers often use insects like ants or beetles as models, providing a natural blueprint for creating efficient, resilient robots and excellent platforms for studying gaits, coordination, and locomotion strategies. Practical applications for these robots are diverse, ranging from search and rescue missions in disaster zones to exploration in deep-sea or planetary environments where their ability to adapt to unknown and uneven surfaces is crucial.

A hexapod robot with underactuated legs, where some of the joints or movements are not directly controlled by actuators, offers advantages such as energy efficiency, lighter weight, and simpler control systems. The system relies on passive elements, such as springs or natural body dynamics, to assist movement and improve stability through adaptation to the environment. While stability can be a challenge due to fewer active controls, these issues can be counteracted by using articulated bodies, which offer enhanced flexibility and maneuverability compared to rigid-body hexapods. Many insects utilize torso articulation for fluid movement, a principle roboticists borrow to create biomimetic robots, including hexapods, with significant advantages in mobility, adaptability, and control [1,2,3,4,5,6].

An articulated main body allows the robot to twist or bend in the middle of sharper turns, aids dynamic balance by shifting the center of mass, and improves terrain adaptability by allowing the front or rear parts to tilt independently (see Table 1). It also facilitates obstacle negotiation, enabling the robot to arch over small barriers or wriggle through tight gaps. The mechanical design often incorporates additional degrees of freedom (DOFs) through mechanisms like single-axis articulation (e.g., AMOS series robots [7,8], SpiceClimber [9], WhegsTM [10,11,12]) or multi-axis designs (e.g., ModPod [13], Hector [14], Dante II [15]), as seen in Table 1.

**Figure 1:**
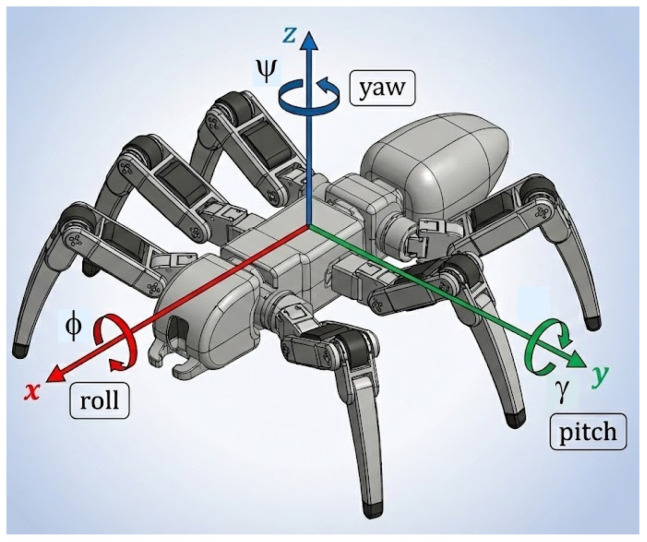
Schematic representation of the robot’s coordinate assignments.

Table 2 summarizes some of the hexapod robots with articulated bodies documented in the literature, presenting each robot’s way of achieving body flexibility, the supplementary DOFs introduced by the design solution used to articulate the body, and the robot’s structural layout. All the robots presented in this table use active articulations to connect the body segments. To describe the DOFs of the articulated body, the robot coordinate system is defined as shown in Figure 1 (AutoCAD 2022). To define the robot’s structural layout, an expression like $n_{1}+n_{2}$ or $n_{1}+n_{2}+n_{3}$ is used, where $n_{i}$ represents the $i^{th}$ segment, starting from the head of the robot, and $n_{i}$ is the number of legs of this segment. “+” represents the connection between segments.

Despite these advancements, a key limitation in the existing designs documented in the literature is that none allow for combined rotations of the body segments around both the longitudinal (roll) and transverse (pitch) axes simultaneously. The approach in this paper, which continues some previous research [19,20,21,22], addresses this critical gap by presenting a novel concept that utilizes a three-segment body connected by two active universal joints, each actuated by four-bar crank-rocker mechanisms. This design not only improves terrain adaptability—allowing the robot to step over obstacles up to 60 mm high and climb slopes up to 30°—but also enables the robot to walk on both sides and autonomously recover from a rollover event, a critical capability in high-risk environments. This paper details a structural synthesis, possible body connection actuation solutions, a robot design, a control algorithm, and experimental results validating this approach.

## 2. Materials and Methods

### 2.1. Structural Synthesis

**Figure 2:**
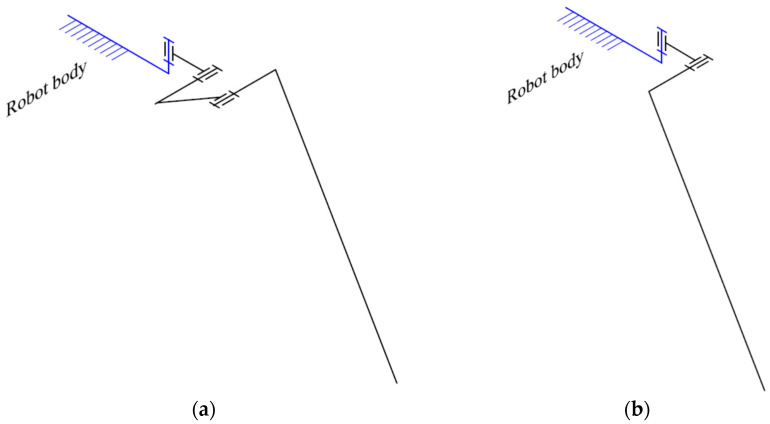
Common open-loop leg mechanisms for hexapod robots: (**a**) Three-DOFs configuration; (**b**) Two-DOFs configuration.

Typically, a complete leg of a hexapod walking robot must have three DOFs, as seen in Figure 2a, but there are also situations where they can have only two such DOFs (Figure 2b). The use of leg mechanisms with more than three DOFs does not provide essential benefits and leads to additional problems.

**Figure 3:**
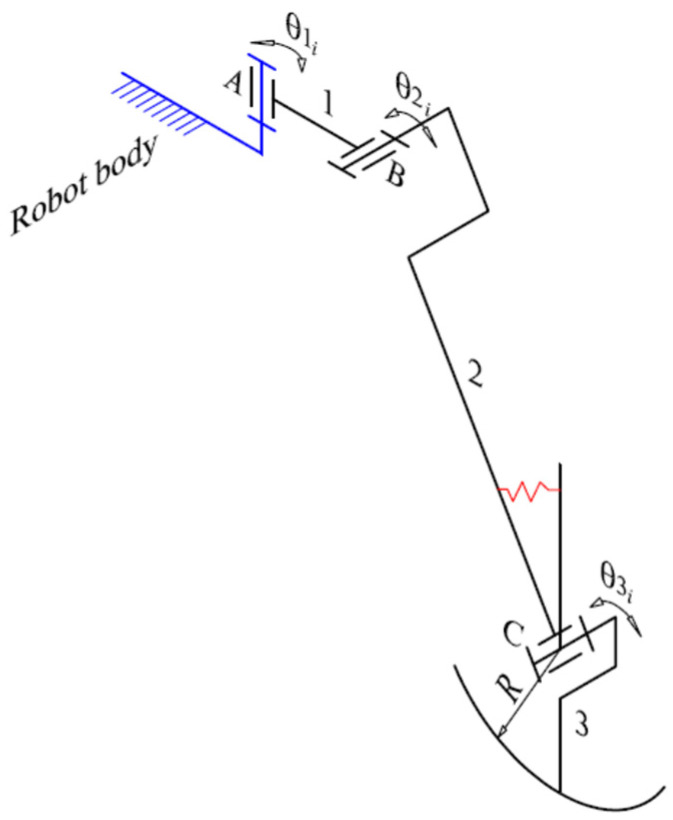
Optimized kinematic architecture of the robotic leg. The notations represent: 1, 2 and 3—links of the leg mechanism; *A* and *B*—actuated joints; *C*—passive joint; *R*—radius of the spherical cap representing the sole of the foot; $\theta_{j_{i}},\;j=1÷3$—angular position parameters of the joints.

Robots with legs with two DOFs require a simpler control system, but they inherently slip a little during walking, which is undesirable from a control point of view and introduces additional mechanical stresses. Considering these issues, and to eliminate the unwanted effects while still using only two actuators per leg, a third passive joint was introduced into the kinematic chain of the leg, a joint “actuated” by a spring (Figure 3). To simplify the control of the robot, the additional link introduced as the sole of the leg is a spherical cap with a radius equal to the length of the respective link.

To improve terrain adaptation and to facilitate rollover recovery, the robot body was designed with three segments connected by two universal joints (with two axes of rotation). Two legs are provided on each body segment.

**Figure 4:**
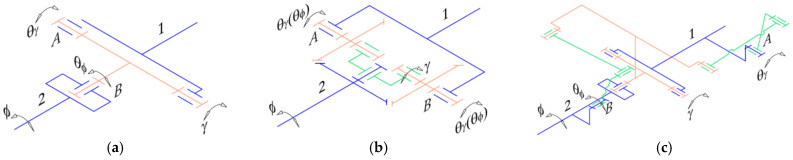
Possible actuation solutions of the universal joint connecting two robot body segments: (**a**) direct-drive configuration—actuators are mounted directly on the primary joint axes; (**b**) gear-driven mechanism—motion is transmitted through a gear mechanism; (**c**) linkage-actuated design—each rotational axis of the universal joint is driven by an independent four-bar mechanism. The notations used in this figure represent: 1 and 2—robot body segments; *A* and *B*—actuated joints; $\theta_{\gamma }$ and $\theta_{\phi }$—angular position parameters of the actuated joints; γ and ϕ—angular position parameters of the pitch and roll joints.

Next, the problem arises of finding the most suitable solution for actuating the body articulations. For this, several options were considered, including mounting actuators directly (Figure 4a) or using gear mechanisms (Figure 4b). However, the kinematic solution that allowed the easiest integration of force sensors for active compliance was the one shown in Figure 4c, which uses two four-bar crank-rocker mechanisms for each universal joint.

**Figure 5:**
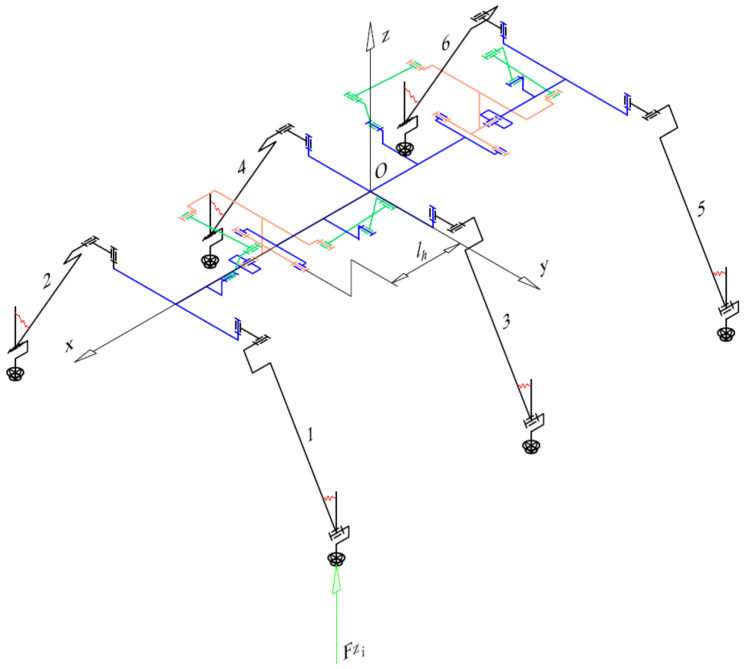
Overall kinematic scheme of the articulated hexapod walking robot, including the leg numbering convention. The notations represent: xyz—coordinate system with its origin O in the body’s geometric center; 1,⋯,6—leg mechanisms; $l_{h}$ horizontal distance between the body’s geometric center and the pitch axis of the universal joint; $F_{z_{i}}$—ground reaction force.

Figure 5 illustrates the overall kinematic diagram of the hexapod walking robot. This configuration enables the chassis to adapt to diverse terrains, specifically allowing the robot—with dimensions of 390 × 170 × 150 mm—to clear obstacles up to 60 mm in height. Furthermore, the simplified leg kinematics allows the robot to scale or descend inclines of up to 30° while minimizing the requirement for additional motors and force sensors. The leg numbering convention is also illustrated in the same figure.

### 2.2. Robot Design and Rollover Capability

**Figure 6:**
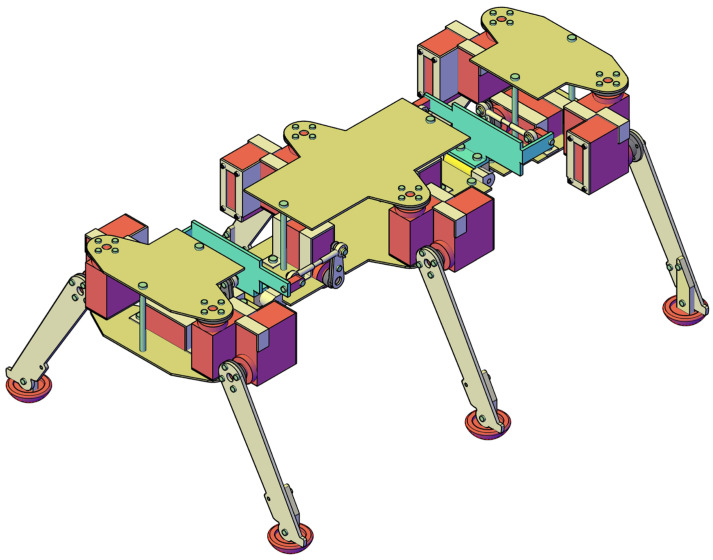
Isometric view illustrating the final assembly of the proposed walking robot.

**Figure 7:**
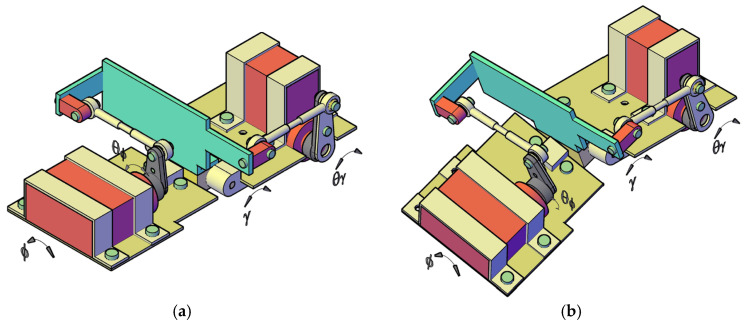
Universal joint assembly connecting two robot body segments: (**a**) coplanar configuration for flat terrain traversal (*θ_γ_* = *θ_ϕ_* = 0); (**b**) multi-planar configuration for navigating uneven terrain (*θ_γ_* ≠ 0; *θ_ϕ_* ≠ 0).

**Figure 8:**
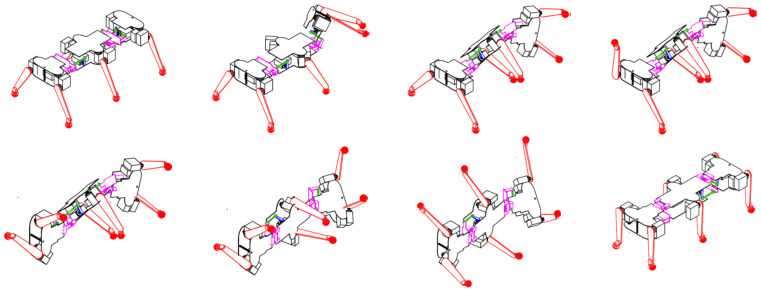
Sequential snapshots of the robot’s rollover maneuver.

In recent years, several biomimetic robots, including walking machines featuring articulated bodies, have been developed [1,7,8,9,10,11,12,13,14,15,16,17,18,23,24]. Building upon the kinematic architecture illustrated in Figure 5, we have designed a similar hexapod (Figure 6). To minimize mechanical complexity, each of the six legs incorporates only two actuated joints. Despite this constrained leg mobility, the integrated architecture achieves superior locomotive versatility and overall performance. The body segmentation and actuated universal joints (Figure 7) provide two primary functions: (i) active suspension and terrain compliance via commanded pitch and roll between segments; and (ii) rollover recovery by executing a predefined sequence to return to an upright posture (Figure 8). The robot can locomote on either side (upright or supine) using the same gait generator with appropriate body-joint references.

While designing a robot capable of transitioning to its back and maintaining locomotion in a supine posture may initially seem impractical, a rigorous analysis reveals its significant utility for extra-terrestrial exploration. During operations on rugged Martian terrain—compounded by the planet’s reduced gravitational acceleration (3.7 m/s^2^)—the risk of overturning is substantial. Leveraging the articulated joints connecting its three chassis segments, the robot exhibits exceptional mobility; it can adapt to irregular topographies and execute seamless transitions between nominal and supine walking modes. This self-righting maneuver is performed by executing an operator-defined motion sequence stored in the onboard flight computer. Sequential snapshots of the rollover maneuver from nominal to supine posture are illustrated in Figure 8.

**Figure 9:**
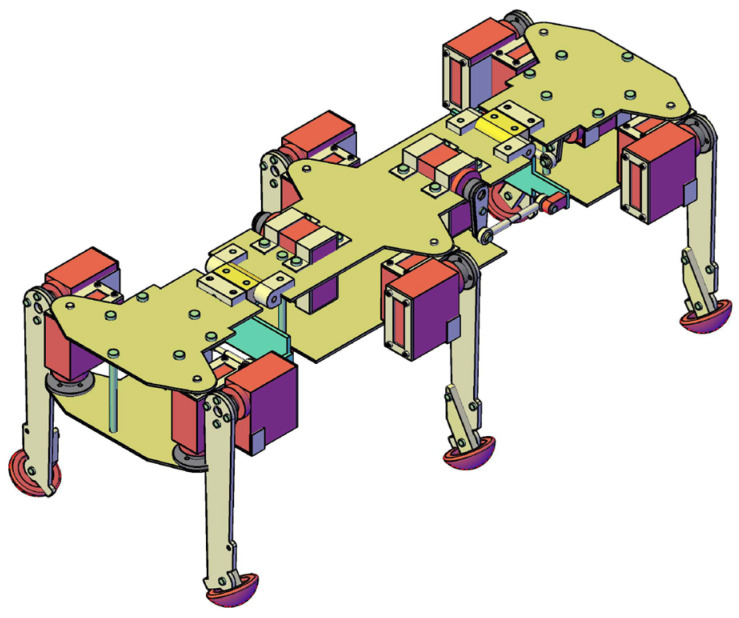
Supine posture of the hexapod robot.

As previously established, the robot is capable of locomotion in both nominal and supine orientations. In principle, this dual-sided mobility could obviate the need for self-righting maneuvers. In practice, however, such transitions remain essential because the scientific instrumentation is not symmetrically distributed across the chassis. Furthermore, in the supine position (Figure 9), the spring-loaded passive joint at the leg tip is rendered ineffective; consequently, the robot experiences intermittent slippage during locomotion. Prolonged travel in this inverted state is therefore not recommended. Instead, the robot should execute a pre-programmed recovery sequence—analogous to the maneuvers depicted in Figure 8—to regain its optimal nominal posture.

### 2.3. Coordinate System, Kinematic Mapping, and Body Joint Limits

#### 2.3.1. Coordinate System

A body-fixed right-handed frame is used, with *x* forward (roll axis), *y* lateral (pitch axis), and *z* upward (yaw axis), as seen in Figure 1. The two actuated DOFs of each universal joint are denoted *γ* (pitch, rotation about the body *y*-axis) and *ϕ* (roll, rotation about the body *x*-axis), as seen in Figure 7.

#### 2.3.2. Actuator-Joint Mapping and Limits from CAD Simulation

**Figure 10:**
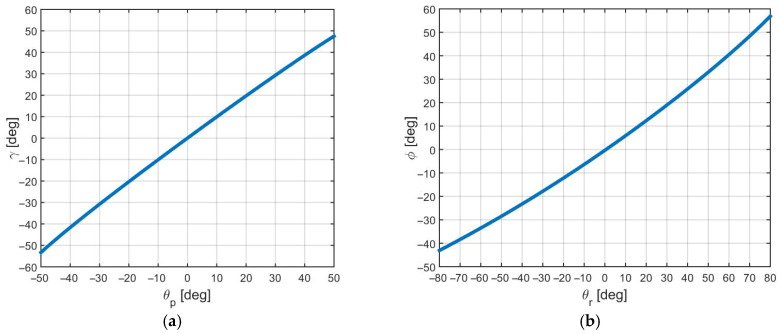
CAD-based kinematic simulation results for the front universal joint: (**a**) pitch motion; (**b**) roll motion.

Actuator-joint mappings were obtained from CAD-based kinematic simulation for the front universal joint (Figure 10). The resulting relations, $\gamma =f_{\gamma }\left(\theta_{p}\right)$ and $\phi =f_{\phi }\left(\theta_{r}\right)$, are smooth and strictly monotonic over the admissible actuator ranges, enabling numerical inversion via lookup tables in the controller (Table 3 and Table 4). The rear joint uses an identical mechanism arranged as a mirror image; therefore, the same mappings apply in magnitude, with an inverted sign convention for the roll DOF.

(1) $$\gamma_{rear}=\gamma_{front},\;\phi_{rear}={-\phi }_{front}$$

To prevent operation near kinematic extremes (where linkage nonlinearities and end-stop interactions can degrade control), conservative controller limits are applied after compliance correction and before PWM output.

### 2.4. Active Compliance and Force Distribution

**Figure 11:**
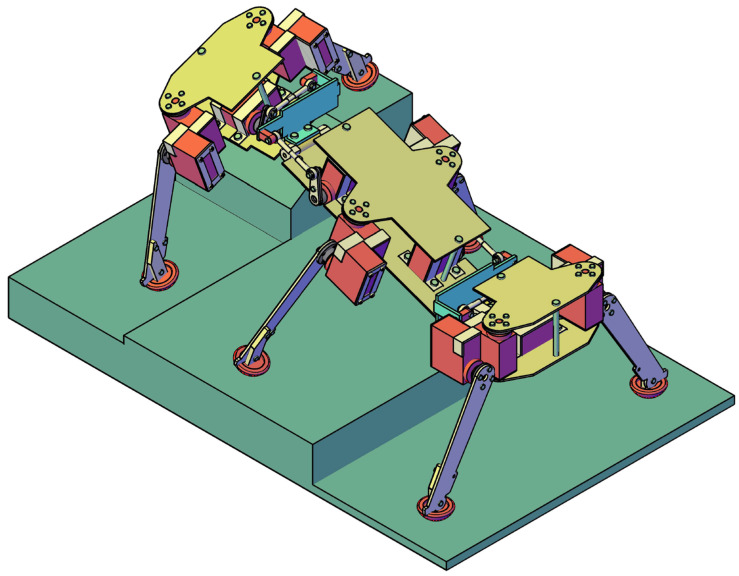
Terrain adaptation capabilities of the articulated robot chassis.

**Figure 12:**
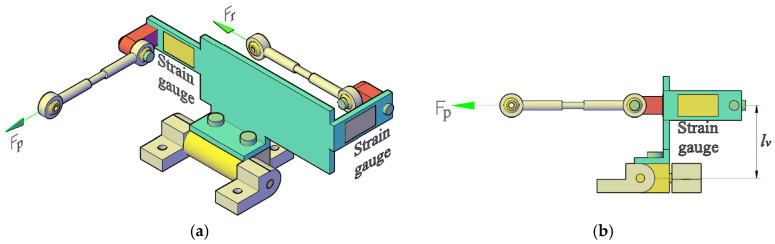
Distribution of measured forces on the common link of the universal joint’s four-bar mechanisms: (**a**) isometric view; (**b**) lateral (side) view.

Controlling such walking machines requires solving the force distribution problem. Numerous studies have addressed foot–ground force distribution using various optimization criteria [11,12,14,23,24,25,26,27,28]. To facilitate terrain adaptation (Figure 11), our design eliminates the need for force sensors at the feet. Instead, strain gauges are mounted on the common link shared by the two four-bar mechanisms that actuate the universal joint (Figure 12).

The primary advantages of this configuration include:

- Reduced actuator count: Minimizing mass and control complexity;
- Localized force sensing: Reducing the total number of transducers required for feedback.

While the overall robot architecture may appear complex, the design requirements are concentrated into two modular subsystems: (i) the leg module, integrating actuators and sensors for the two active degrees of freedom (DOFs); and (ii) the active universal joint, which houses the actuators and force sensing elements.

Although the compliance law can be interpreted as a torque–deflection equation, the prototype does not measure joint torque directly. Instead, strain gauges mounted on the common link of the actuation mechanisms (Figure 12) provide force measurements in N, which are also the quantities reported in Section 4. Therefore, we implement the active compliance outer loop in force form. So, for each degree of freedom of the universal joint, we may consider the general equation

(2) $$\left(\theta^{ref}-\theta \right)-c\left(F^{ref}-F\right)=0,$$

where

- *F*, in N, and *θ*, in deg, denote the current (measured) force and angular position for the pitch or roll joint, respectively;
- $F^{ref}$ and $\theta^{ref}$ are the corresponding reference (estimated) values;
- c represents the system compliance in deg/N (equivalently, K=1/c  is a virtual stiffness in N/deg).

When expressing the same law in torque form,

(3) $$\left(\theta^{ref}-\theta \right)-c_{\tau }\left(\tau^{ref}-\tau \right)=0,$$

the equivalent torque is approximated by $\tau \approx Fl_{v}$, with the system compliance $c_{\tau }$ in deg/Nm in that case.

The following section details the computation of the reference force ($F^{ref}$). Initially, the force distribution for a specific gait state is derived from the static equilibrium equations of the entire system. To simplify the formulation, the following assumptions are made:

- Negligible Dynamics: The robot’s velocity and mass are sufficiently low to ignore inertial effects;
- Vertical Interaction: Ground reaction forces are assumed to act exclusively in the vertical direction (Figure 5);
- Mass Distribution: The mass of the segments formed by links 2 and 3 (Figure 3) is considered negligible relative to the actuators at joints *A* and *B*. These actuators are rigidly coupled, forming link 1 (Figure 3). Unlike previous studies where the total leg mass is often discounted, our design accounts for the combined mass of the six legs, as it is comparable to the main chassis mass. Consequently, the inertial influence of the leg actuators is explicitly included in the equilibrium model as the mass of the entire leg.

Scope clarification: The vertical-only assumption is used to generate a consistent reference distribution for slow walking on moderately uneven terrain. On laterally sloped terrain, during slip, or when tangential contact forces become significant, the model will underestimate coupling effects and can reduce the accuracy of predicted internal loads; this limitation motivates future work that includes tangential contact components and friction constraints.

Assuming the robot’s body mass ($m_{b}$), its center of mass ($r_{b}\left(x_{b},y_{b}\right)$), the individual leg mass ($m_{l}$), and the respective leg center-of-mass positions ($r_{l_{i}}\left(x_{l_{i}},y_{l_{i}}\right)$) are known—and further assuming mass symmetry across all six legs—the following equilibrium relationship is established:

(4) $$\left\{\begin{array}{l} G=\left(m_{b}+6\cdot m_{l}\right)\cdot g\\ G\cdot x_{c}=(m_{b}\cdot x_{b}+m_{l}\cdot \sum_{i=1}^{6}x_{l_{i}})\cdot g\\ G\cdot y_{c}=(m_{b}\cdot x_{b}+m_{l}\cdot \sum_{i=1}^{6}y_{l_{i}})\cdot g \end{array}\right.,$$

where:

- *G* denotes the total vehicle weight (expressed in N);
- i expresses the leg indices (i=1,…,6);
- $\left(x_{l_{i}},y_{l_{i}}\right)$ denote the coordinates of the center of mass for leg *i*;
- $\left(x_{c},y_{c}\right)$ denote the coordinates of the robot’s global center of mass;
- $\left(x_{b},y_{b}\right)$ denote the coordinates of the robot’s geometric center;
- $m_{b}$ is the robot body mass;
- $m_{l}$ represents the mass of a single leg assembly;
- g is the gravitational acceleration.

Due to the robot’s inherent symmetry, the body’s geometric center coincides with the origin of the reference frame; therefore, $x_{b}=y_{b}=0$, and

(5) $$\left\{\begin{array}{l} G=\left(m_{b}+6\cdot m_{l}\right)\cdot g\\ G\cdot x_{c}=m_{l}\cdot g\cdot \sum_{i=1}^{6}x_{l_{i}}\\ G\cdot y_{c}=m_{l}\cdot g\cdot \sum_{i=1}^{6}y_{l_{i}} \end{array}\right..$$

If $\theta_{1_{i}}$ and $\theta_{2_{i}}$ are known (refer to Figure 3), the individual leg center-of-mass positions can be computed at any given time for i=1,…,6, and consequently the overall system center of mass is derived as follows:

(6) $$\left\{\begin{array}{c} x_{c}=\frac{\sum_{i=1}^{6}x_{l_{i}}}{\frac{m_{b}}{m_{l}}+6}\\ y_{c}=\frac{\sum_{i=1}^{6}y_{l_{i}}}{\frac{m_{b}}{m_{l}}+6} \end{array}\right..$$

Assuming that the ground reaction forces act exclusively in the vertical direction, the following equations are obtained:

(7) $$\left\{\begin{array}{l} \sum_{i\in I}F_{z_{i}}=G\\ \sum_{i\in I}F_{z_{i}}\cdot x_{i}=G\cdot x_{c}\\ \sum_{i\in I}F_{z_{i}}\cdot y_{i}=G\cdot y_{c} \end{array}\right.,$$

where:

- *I* denotes the set of supporting legs; depending on the gait type and its specific phase, this set typically includes between three and six legs in active ground contact;
- (*x_i_*, *y_i_*), for *i =* 1, …, 6, represent the foot-tip coordinates relative to the origin of the reference frame (the robot’s geometric center).

Equation (7) can be rewritten as follows:

(8) $$A\cdot F_{z}=G,$$

where

(9) $$A=\left[\begin{array}{cccccc} 1 & 1 & 1 & 1 & 1 & 1\\ x_{1} & x_{2} & x_{3} & x_{4} & x_{5} & x_{6}\\ y_{1} & y_{2} & y_{3} & y_{4} & y_{5} & y_{6} \end{array}\right],\;F_{z}=\left[\begin{array}{c} F_{z_{1}}\\ F_{z_{2}}\\ F_{z_{3}}\\ F_{z_{4}}\\ F_{z_{5}}\\ F_{z_{6}} \end{array}\right],\;G=\left[\begin{array}{c} G\\ m_{l}\cdot g\cdot \sum_{i=1}^{6}x_{l_{i}}\\ m_{l}\cdot g\cdot \sum_{i=1}^{6}y_{l_{i}} \end{array}\right].$$

Note on Assumption: While the assumption of purely vertical ground reaction forces limits the model’s validity for highly uneven or slippery terrain (contradicting the paper’s full application scope), it provides a necessary simplification for the initial static analysis and a baseline for our experimental comparison. Future models will relax this assumption to include tangential friction forces.

Whenever more than three legs are in contact with the ground, the system (8) becomes underdetermined (i.e., it contains more unknowns than equations). In this case, the minimum-norm solution is given by:

(10) $$F_{z}=A^{+}\cdot G,$$

where $A^{+}=A^{T}{\left(A\cdot A^{T}\right)}^{-1}$ denotes the Moore–Penrose pseudoinverse of matrix A.

Note on Pseudoinverse: The application of the Moore–Penrose pseudoinverse solution minimizes the sum of the squares of the vertical forces ($J=\sum F_{z_{i}}^{2}$) while satisfying the static-equilibrium equality constraints. The numerical conditioning of the matrix A is highly dependent on the leg configuration and gait phase. In typical hexapod gaits where the supporting legs form a stable polygon, the solution is generally robust. However, sensitivity to extreme leg configurations or very narrow support polygons is a known limitation that warrants consideration in dynamic scenarios.

Once the vertical force distribution is known, the reference forces for the two DOFs of the universal joints can be determined by enforcing rotational equilibrium about the corresponding axes. An exact estimate of the reference torque is not required; the active compliance law in (2) can equivalently be implemented by setting $F^{ref}=0$, which allows the joints to passively follow the terrain dynamics subject to the defined compliance c (or stiffness K).

Given the theoretical contact forces and the foot-tip coordinates relative to the robot’s center, the forces acting on the universal joints can be determined for each degree of freedom. The proposed model is an approximation, as it neglects reaction forces within the kinematic chain and accounts exclusively for gravitational loads. To derive the force for the mechanism facilitating flexion–extension (pitch motion), we refer to the configurations shown in Figure 5 and Figure 12. From the static equilibrium of forces, it follows that:

- for the front-mounted mechanism,

(11) $$F_{p_{f}}=-\frac{F_{z_{1}}\cdot \left(x_{1}-l_{h}\right)+F_{z_{2}}\cdot \left(x_{2}-l_{h}\right)}{l_{v}},$$

- for the rear joint actuation mechanism,

(12) $$F_{p_{b}}=-\frac{F_{z_{5}}\cdot \left(-x_{5}-l_{h}\right)+F_{z_{6}}\cdot \left(-x_{6}-l_{h}\right)}{l_{v}}.$$

To analyze the pronation–supination (roll motion), the same figures are considered. By applying the moment equilibrium conditions, we obtain:

- for the front-mounted mechanism,

(13) $$F_{r_{f}}=\frac{F_{z_{1}}\cdot y_{1}-F_{z_{2}}\cdot y_{2}}{l_{v}},$$

- for the rear joint actuation mechanism,

(14) $$F_{r_{b}}=\frac{F_{z_{5}}\cdot y_{5}-F_{z_{6}}\cdot y_{6}}{l_{v}}.$$

One of the following sections presents several plots illustrating the variations in joint reference forces (utilized for active compliance) across different gait patterns.

## 3. Control Architecture

**Figure 13:**
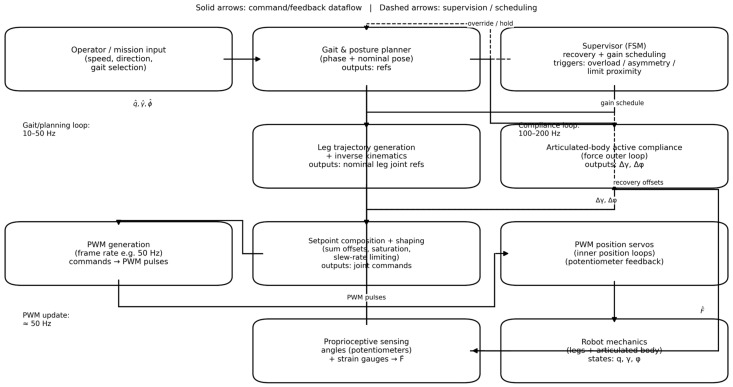
Overall control architecture of the hexapod robot (overview). Nominal gait/posture references are shaped and sent to PWM position servos. Proprioceptive feedback (potentiometer angles and strain-gauge loads) supports a supervisory FSM and an articulated-body force-based compliance outer loop (detailed in Figure 14).

**Figure 14:**
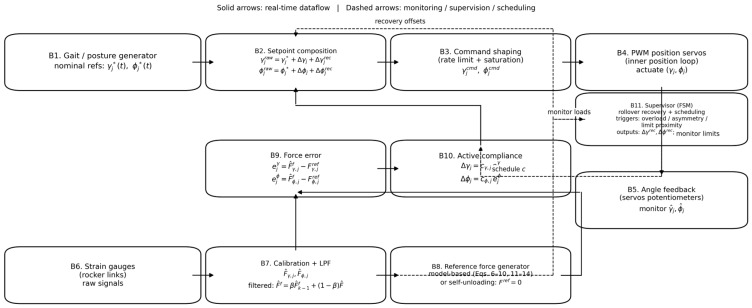
Force-based active compliance outer loop around PWM position servos. Strain-gauge loads are calibrated and filtered to obtain $\hat{F}$, compared with reference forces $F^{ref}$, and mapped to pitch/roll corrections (∆γ, ∆ϕ). Corrections are summed with nominal references and recovery offsets, then shaped (saturation and slew-rate limiting) before PWM output; dashed links indicate supervision/scheduling signals.

This section details the real-time control architecture used to generate articulated-body pitch/roll motion and to implement force-based active compliance around PWM position servos. The presentation is organized from the system-level control stack (Figure 13) to the detailed compliance loop (Figure 14) and its discrete-time execution (Algorithm 1).

### 3.1. Sensing and Actuation Constraints

The prototype relies exclusively on proprioceptive sensing: (i) strain gauges mounted on the common rocker links of the universal-joint actuation mechanisms, and (ii) joint angle feedback provided by the internal potentiometers of the PWM position servos. No IMU, foot force sensors, or exteroceptive sensors are used. Consequently, interaction regulation is implemented as an outer-loop setpoint modulation that wraps the embedded servo position controllers.

The inner servo loops track commanded angles at the PWM update rate, while the proposed compliance controller updates the reference angles at an outer-loop sampling period $T_{s}$. Because the PWM frame rate limits how frequently new setpoints are applied by the servos, saturation and slew-rate limiting are applied to all commanded angles to avoid end-stop impacts and to keep the commanded motion smooth under changing interaction loads.

### 3.2. System-Level Control Stack

Figure 13 summarizes the overall control stack. A gait/posture generator produces nominal references for both the leg joints and the articulated-body universal joints (pitch *γ* and roll *ϕ*). The active compliance outer loop modifies the nominal universal-joint references based on the filtered force-related measurements from the strain gauges, yielding setpoint corrections that are then shaped (rate-limited and saturated) before being converted into PWM commands for the servos.

A supervisory finite-state machine (FSM) monitors proprioceptive signals to detect persistent overload, roll-load asymmetry, and proximity to conservative joint limits. When necessary, the supervisor schedules compliance gains and injects recovery offsets/sequences (e.g., for rollover recovery or limit avoidance). The detailed compliance loop that converts strain-gauge loads into pitch/roll setpoint corrections is shown in Figure 14 and formalized in Algorithm 1.

### 3.3. Force-Based Active Compliance Law and Setpoint Generation

As mentioned before, because the strain gauges provide force-related measurements and the experimental validation is reported in forces, the compliance law is implemented in force form to ensure dimensional clarity. For each universal joint $j\in \left\{front,rear\right\}$ and each DOF $\theta \in \left\{\gamma \;\left(pitch\right),\phi \;(roll)\right\}$, let ${\hat{F}}_{j}^{\theta ,f}$ denote the filtered measured force component, in N, $F_{j}^{\theta ,ref}$ the reference force, in N, and $c_{\theta ,j}$ the compliance gain with units deg/N. The outer-loop compliance correction is

(15) $${\Delta \theta }_{j}=s_{\theta ,j}c_{\theta ,j}\left({\hat{F}}_{j}^{\theta ,f}-F_{j}^{\theta ,ref}\right),\;\;\;\;\;\;\;\;\;\;\;\theta \in \left\{\gamma ,\phi \right\},$$

where $s_{\theta ,j}\in \left\{+1,-1\right\}$ is an optional sign variable used to compensate for effective corrective-direction reversal on steep slopes or off-nominal contacts (see Section 3.4). The commanded setpoint is formed by setpoint composition and command shaping:

(16) $${\;\theta_{j}^{raw}=\theta_{j}^{*}+\Delta \theta }_{j}+\Delta \theta_{j}^{rec},\;{\;\;\;\;\theta }_{j}^{cmd}=shape\left(sat\left(\theta_{j}^{raw},\theta_{min},\theta_{max}\right)\right),$$

where $\Delta \theta_{j}^{rec}$ denotes supervisor recovery offsets, sat(·) enforces conservative joint limits, and shape(·) implements slew-rate limiting (and optional smoothing) prior to PWM output.

Notation: Discrete-time signals are indexed by k (sample index) with sampling period $T_{s}$. The implemented compliance is the force-form gain c (reported in deg/N), while the torque-form interpretation uses $c_{\tau }$ (reported in deg/Nm). To avoid ambiguity with the discrete-time index k in Algorithm 1, the corresponding virtual stiffness is denoted as K=1/c (and $K_{\tau }=1/c_{\tau }$ when needed).

Dimensional Clarification (Torque-Form vs Force-Form): If a torque-form compliance is preferred, Equation (3) can be written as $∆\theta =c_{\tau }\left(\tau -\tau^{ref}\right)$, where $c_{\tau }$ has units deg/Nm. Using an effective lever arm $l_{v}$ such that $\tau \approx Fl_{v}$, the equivalent force-form gain in Equation (15) is $c=c_{\tau }l_{v}$, in deg/N. This keeps the implementation consistent with the measured strain-gauge force quantity.

### 3.4. Reference Forces, Supervision and Recovery Execution

Reference forces are generated in two practical modes. In the model-based mode, a quasi-static force distribution is computed for the current support configuration and mapped to the forces expected in the instrumented rocker links, yielding $F_{j}^{\theta ,ref}$. In the self-unloading mode, $F_{j}^{\theta ,ref}$ is set to zero, producing compliance that reduces sustained internal loading without requiring an accurate load model.

The supervisory FSM monitors (i) overload events in $\left|{\hat{F}}^{f}\right|$, (ii) roll-load asymmetry between joints, and (iii) proximity to conservative joint limits (with angular margins). To avoid chatter, triggers are validated using dwell counters and hysteresis. When the supervisor enters a recovery set, it injects predefined offsets/sequences and schedules the compliance gains (e.g., temporarily stiffer during stabilization and more compliant during settling).

Because no IMU is used, rollover recovery is executed as a deterministic sequence driven by joint angles and load thresholds rather than by explicit body-attitude estimation. For steep descent or laterally sloped contacts, tangential components may reduce or reverse the effective torque induced by the measured load. In such cases, a rule-based sign switch $s_{\theta ,j}$ can be activated when a persistent inconsistency is detected, with hysteresis to prevent rapid toggling.

### 3.5. Compliance-Loop Implementation and Algorithm

Figure 14 details the implementation of the compliance outer loop around PWM position servos. Solid arrows indicate the real-time dataflow, whereas dashed arrows indicate monitoring/scheduling signals used by the supervisor. Algorithm 1 summarizes the discrete-time execution order implemented in software. Load filtering is implemented as a first-order IIR low-pass filter:

(17) $${\hat{F}}^{f}\left[k\right]=\beta {\hat{F}}^{f}\left[k-1\right]+\left(1-\beta \right)\hat{F}\left[k\right],\;\;\;\;\;0<\beta <1.$$

Notation: The hat symbol $\left(\hat{\cdot }\right)$ denotes sensor-based force estimates obtained from strain-gauge signals through calibration; the superscript f denotes the low-pass filtered estimate used by the controller. Thus, $F^{raw}$ is the raw strain-gauge signal, $\hat{F}$ is the calibrated force estimate, in N, and ${\hat{F}}^{f}$ is the filtered estimate. The force tracking error is computed as $e={\hat{F}}^{f}-F^{ref}$.

**Algorithm 1.** Force-based active compliance outer loop (PWM position servos).**Inputs (at step *k*).** Nominal references $\gamma_{j}^{*}\left[k\right]$, $\phi_{j}^{*}\left[k\right]$; strain-gauge signals; measured angles $\gamma_{j}\left[k\right]$, $\phi_{j}\left[k\right]$ (servo potentiometers); support/state information (optional, for model-based $F^{ref}$.) **Parameters.** Outer-loop period $T_{s}$; LPF coefficient β; compliance gains $c_{j}^{\gamma }$, $c_{j}^{\phi }$, in deg/N; thresholds $F_{th}$; dwell counters $N_{dwell}$, $N_{exit}$; limit margins $\theta_{marg}$; saturation limits $\left[\gamma_{min},\gamma_{max}\right]$,$\left[\phi_{min},\phi_{max}\right]$; slew limits ${∆\gamma }_{max}$, ${∆\phi }_{max}$ (and/or a generic ${∆\theta }_{max}$). **Outputs.** Commanded setpoints $\gamma_{j}^{cmd}\left[k\right]$, $\phi_{j}^{cmd}\left[k\right]$ (converted to PWM pulses at the servo frame rate). **Initialization (once).** Set filtered forces ${\hat{F}}_{j}^{\theta ,f}\left[0\right]=0$; $s_{j}^{\theta }=+1$; set FSM state = NORMAL and counters = 0; set $\gamma_{j}^{cmd}\left[0\right]=\gamma_{j}^{*}\left[0\right]$, $\phi_{j}^{cmd}\left[0\right]=\phi_{j}^{*}\left[0\right]$. **At each outer-loop update (every**
$T_{s}$**), for each joint *j* and** $\theta \in \left\{\gamma ,\;\phi \right\}$:
1.Acquire strain-gauge signals and apply calibration to obtain raw forces $F_{j,raw}^{\theta }\left[k\right]$ (N).
2.Low-pass filter:
(18)$${\hat{F}}_{j}^{\theta ,f}\left[k\right]\leftarrow \beta {\hat{F}}_{j}^{\theta ,f}\left[k-1\right]+\left(1-\beta \right)F_{j,raw}^{\theta }\left[k\right].$$
3.Compute reference forces $F_{j}^{\theta ,ref}\left[k.\right]$ using either (a) model-based force distribution mapped to the instrumented links, or (b) self-unloading with $F^{ref}=0$.4.Compute force errors:(19)$$e_{j}^{\theta }\left[k\right]\leftarrow {\hat{F}}_{j}^{\theta ,f}\left[k\right]-F_{j}^{\theta ,ref}\left[k\right].$$5.Supervisor (FSM): update dwell/hysteresis counters; detect overload/asymmetry and proximity to limits using $\theta_{marg}$. If triggered, enter RECOVERY and schedule gains $c_{j}^{\theta }\left[k\right]$ and recovery offsets ${∆\theta }_{j}^{rec}$; if stable for $N_{exit}$, return to NORMAL.
6.Optional sign management: if persistent wrong-way compliance is detected (e.g., steep slope), toggle $s_{j}^{\theta }$ with hysteresis to compensate for effective torque reversal.7.Compute compliance correction:
(20)$$∆\theta_{j}\left[k\right]\leftarrow s_{j}^{\theta }c_{j}^{\theta }\left[k\right]e_{j}^{\theta }\left[k\right].$$
8.Setpoint composition:
(21)$$\gamma_{j}^{raw}\left[k\right]\leftarrow \gamma_{j}^{*}\left[k\right]+∆\gamma_{j}\left[k\right]+∆\gamma_{j}^{rec}\left[k\right],\;\;\;\;\;\phi_{j}^{raw}\left[k\right]\leftarrow \phi_{j}^{*}\left[k\right]+∆\phi_{j}\left[k\right]+∆\phi_{j}^{rec}\left[k\right].$$
9.Saturation:
(22)$$\gamma_{j}^{sat}\left[k\right]\leftarrow sat\left(\gamma_{j}^{raw}\left[k\right],\;\gamma_{min},\gamma_{max}\right),\;\;\;\;\;\phi_{j}^{sat}\left[k\right]\leftarrow sat\left(\phi_{j}^{raw}\left[k\right],\;\phi_{min},\phi_{max}\right).$$
10.Slew/rate limiting (and optional smoothing):(23)$\gamma_{j}^{cmd}\left[k\right]\leftarrow \gamma_{j}^{cmd}\left[k-1\right]+sat\left(\gamma_{j}^{sat}\left[k\right]-\gamma_{j}^{cmd}\left[k-1\right],\;{-∆\gamma }_{max},{+∆\gamma }_{max}\right),$
(24)$$\phi_{j}^{cmd}\left[k\right]\leftarrow \phi_{j}^{cmd}\left[k-1\right]+sat\left(\phi_{j}^{sat}\left[k\right]-\phi_{j}^{cmd}\left[k-1\right],\;{-∆\phi }_{max},{+∆\phi }_{max}\right).$$
11.PWM update: convert $\gamma_{j}^{cmd}\left[k\right],\;\phi_{j}^{cmd}\left[k\right]$ to the PWM pulses; if $T_{s}$ is faster than the servo frame rate, hold the most recent shaped command until the next PWM update.

At each outer-loop update $T_{s}$, the controller (i) acquires and filters strain-gauge loads, (ii) computes $F^{ref}$, (iii) computes force errors and compliance corrections $\left(∆\gamma ,\;∆\phi \right)$, (iv) composes setpoints with any supervisory recovery offsets, and (v) applies saturation and slew-rate limiting before PWM output. In the reported experiments, the outer-loop compliance update rate was selected to be higher than the PWM frame rate to improve disturbance rejection while accounting for the fact that new PWM setpoints are only applied at the servo update rate. The baseline parameter set (filtering, limits, compliance gains, thresholds, and dwell times) should be reported in a dedicated table to support reproducibility.

### 3.6. Baseline Control Parameters (Reproducibility)

Table 5 reports a baseline parameter set used for the experiments (loop rates, filtering, compliance gains, thresholds, conservative joint limits, and slew-rate limits), enabling reproducibility of the control implementation.

## 4. Results

This section presents the experimental results obtained from the hexapod prototype, based on the overall kinematic scheme in Figure 5 and the 3D CAD concept in Figure 6. These initial findings are presented as a proof-of-concept demonstration of the robot’s capabilities on various surfaces rather than a statistically rigorous analysis.

### 4.1. The Experimental Prototype and Scope

**Figure 15:**
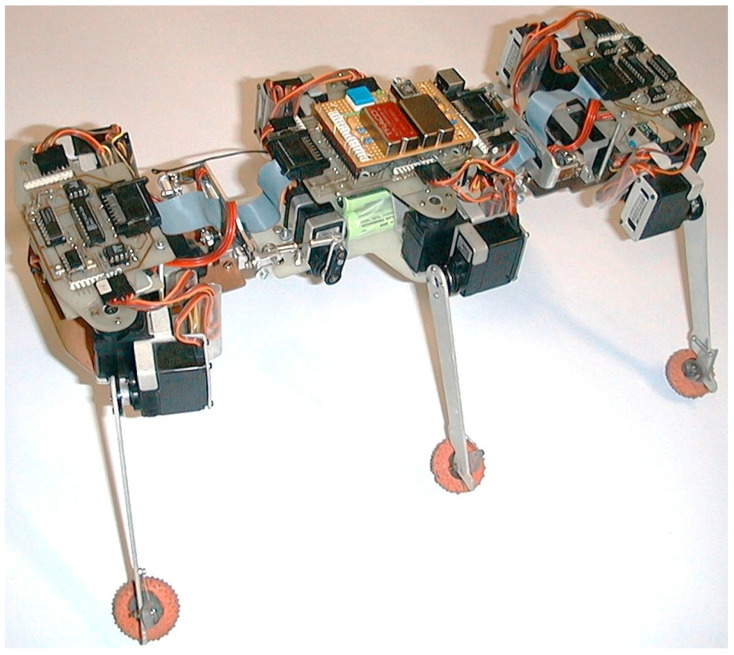
Physical prototype of the developed hexapod walking robot.

The physical prototype of the robot is presented in Figure 15. The robot has overall dimensions of 390 × 170 × 150 mm and can step over obstacles with a height of up to 60 mm and climb or descend an inclined plane with an angle of up to 30°. Thanks to the servo-controlled universal joints, the robot can locomote on both sides and autonomously reorient, enabling recovery from rollover. The experimental results that follow are from selected tests and do not include statistical analysis, repeatability metrics, or uncertainty on the bounds, representing preliminary findings of the concept’s viability.

### 4.2. Measured Forces in the Universal Joints and Estimation Method

Theoretical (simulation) and experimental data were collected regarding the forces acting on the front and rear universal joints during pitch and roll movements. The theoretical loads on the strain gauges were estimated using simplified force equilibrium equations (Equations (11)–(14)), which rely on specific lever-arm assumptions.

Note on force estimation: These force estimation equations assume a simplified, static configuration and rely on nominal lever-arm lengths. They were used for initial validation in expected (nominal) configurations. Full experimental validation for off-nominal or extreme configurations was outside the scope of these initial tests but is a critical area for future work.

#### 4.2.1. Wave Gait

**Figure 16:**
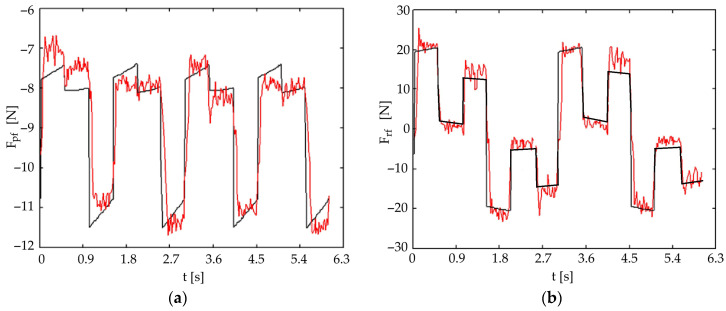
Comparison of theoretical (black) and experimental (red) forces in the front universal joint for a wave gait (five-leg support phase): (**a**) pitch motion; (**b**) roll motion.

**Figure 17:**
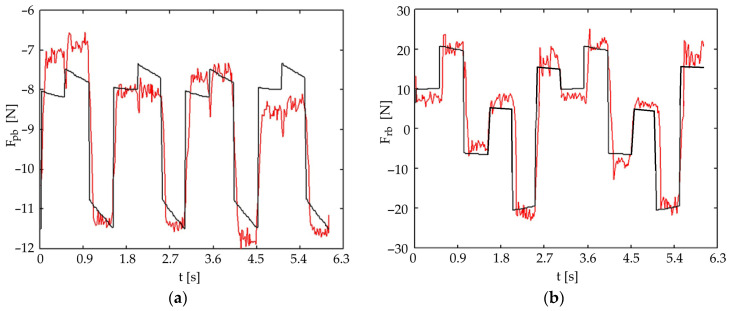
Comparison of theoretical (black) and experimental (red) forces in the rear universal joint for a wave gait (five-leg support phase): (**a**) pitch motion; (**b**) roll motion.

Figure 16 presents the theoretical (black line) and experimental (red line) forces in the front universal joint for a wave gait with five legs in contact with the ground. Figure 17 presents the same forces for the rear universal joint. It was observed that pitch-joint strain-gauge forces are estimated more accurately. The roll forces show greater experimental variation compared to the theoretical model, highlighting the need for active control strategies.

#### 4.2.2. Symmetrical Tripod Gait

**Figure 18:**
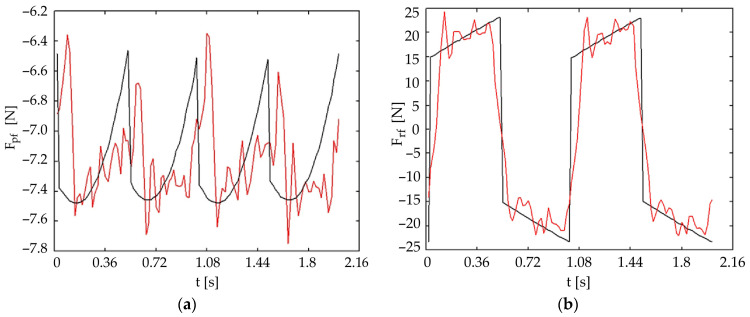
Comparison of theoretical (black) and experimental (red) forces in the front universal joint during a tripod gait (three-leg support phase): (**a**) pitch motion; (**b**) roll motion.

**Figure 19:**
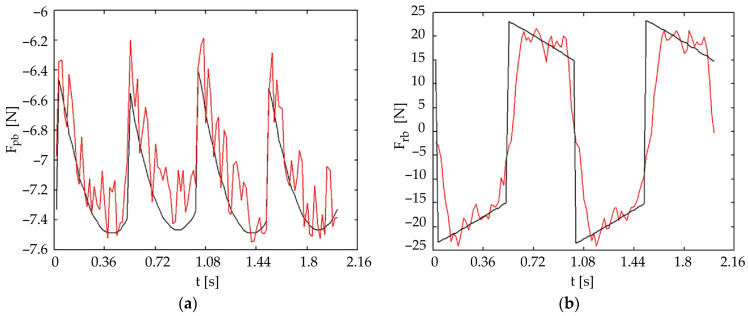
Comparison of theoretical (black) and experimental (red) forces in the rear universal joint during a tripod gait (three-leg support phase): (**a**) pitch motion; (**b**) roll motion.

Figure 18 and Figure 19 present the same forces for a symmetrical tripod gait (with three legs in contact with the ground). In this scenario, the roll-joint forces are also well estimated, indicating better model applicability for this specific, more stable gait.

Note on Repeatability and Uncertainty: The plots shown correspond to representative trials; repeated synchronized time-series datasets for statistical aggregation were not retained. Future work will include repeated trials across terrain classes with uncertainty bounds computed from calibration residuals, steady-state noise, and cross-trial variability.

### 4.3. Rollover Recovery Sequences

The robot is capable of transitioning from the normal position to the supine position and vice versa, allowing it to right itself in case of overturning. Several snapshots of the motions performed during the rollover, similar to those used during the trials, are shown in Figure 8. This demonstrates the functional success of the design’s key feature.

## 5. Discussion

### 5.1. Interpretation of Data and Concept Validation

The experimental results, which compare theoretical simulations with physical prototype data, confirm the overall functional validity of the proposed concept, particularly the robot’s ability to adapt to terrain irregularities and recover from rollover. The measured forces were generally close to the theoretical ones in nominal configurations, supporting the core hypothesis.

Table 3 and Table 4 provide a direct quantitative link between mechanism geometry (CAD simulation) and control implementation (saturation bounds and compliance margins). The monotonic mapping supports lookup-table inversion and clarifies the allowable body articulation used throughout the control pipeline.

Pitch Force Accuracy and Model Assumptions: The striking resemblance between the theoretical and experimental forces for pitch movements (Figure 16a, Figure 17a, Figure 18a and Figure 19a) indicates that the initial simplified static model of the robot, which assumes ground reaction forces act only in a vertical direction, is sufficiently accurate for predicting these forces under nominal conditions. This simplification effectively reduces the complexity of the initial control design.

Roll Force Variation and Dynamic Considerations: The greater variation observed in the experimental roll forces (Figure 16b and Figure 17b), even if better estimated in the symmetric tripod gait case (Figure 18b and Figure 19b), suggests an increased sensitivity of the roll movement to gait dynamics and the foot-ground interaction. This validates the necessity of an active control approach using force sensors to ensure dynamic stability, as relying purely on passive dynamics or simplified static models proves insufficient for highly accurate trajectory control in all scenarios.

Terrain Adaptability as Proof of Concept: The functional success in stepping over 60 mm obstacles and climbing 30° slopes, capabilities essential in environments where wheeled or tracked robots fail, demonstrates the improved flexibility and maneuverability granted by the body articulations. This performance serves as strong initial evidence for the effectiveness of the design approach, even within the scope of a preliminary study that lacked formal statistical analysis or repeatability metrics.

### 5.2. Contribution to the Field and Comparison with Prior Work

Gap Addressed: The current design addresses a notable gap in the literature; as previously mentioned, none of the existing designs of hexapod robots with an articulated body allowed for robust, combined rotations around both the *x* and *y* axes (roll and pitch). Our approach provides this combined movement, enabling a higher degree of body conformity to the environment.

Survival Advantage: The ability to reorient and walk in a supine position provides a critical resilience advantage, especially for planetary exploration missions such as those on Mars, where the lower gravity increases the risk of overturning. This dual-locomotion capability can prevent mission failure in a high-risk scenario.

Underactuation/Articulation Trade-off: The approach successfully combines the reduced complexity of two DOFs underactuated legs with the flexibility of the three-segment articulated body. This balance ensures robust performance on variable terrain while maintaining a relatively simple overall system design with fewer actuators and fewer locations for force measurement.

### 5.3. Current Limitations and Future Directions

Supine Movement: A key limitation is the reduced performance in the supine (back-walking) position, where the passive spring-loaded leg joint no longer functions as intended, leading to minor slippages. Long-distance travel in this position is not recommended; the priority remains self-righting.

Model Simplification and Rigor: The current model is approximate, as it neglects tangential ground reaction forces and the dynamics of the kinematic pairs. Furthermore, the reliance on the Moore–Penrose pseudoinverse solution can be sensitive to numerical conditioning in extreme leg configurations.

Future Research: Future work must focus on the practical implementation of advanced control strategies, such as fuzzy logic adaptive control, to better manage dynamic variations and ensure more autonomous decision-making. Future experimental procedures will be expanded to include rigorous statistical analysis, repeatability studies, and testing on a wider variety of textured surfaces beyond simple slopes and obstacles to ensure robustness in genuinely unstructured environments. The analytical model will also be refined to incorporate more comprehensive force dynamics.

## 6. Conclusions

In this paper, a novel concept for a hexapod walking robot designed for enhanced adaptability on rough terrain was presented in detail. The core contribution is the integration of an articulated body, consisting of three segments connected by two actively controlled universal joints, which allows for combined pitch and roll movements not found in most existing designs. This mechanical solution, combined with underactuated legs that use a passive spring element, allows the robot to achieve a balance between mechanical simplicity and robust performance on variable terrain.

The feasibility and effectiveness of the design were validated through extensive experimental tests on a physical prototype. The measured forces in the joints closely matched the theoretical simulations for nominal pitch movements, confirming the validity of the initial simplified static model as a baseline.

The experimental results demonstrated several key advantages, serving as a proof of concept: the robot can navigate over obstacles up to 60 mm high and traverse inclined planes up to 30° with high stability; the design ensures that desired contact forces are reached quickly, minimizing slippage during locomotion in stable gaits; the autonomous recovery sequences were successfully executed, confirming the robot’s resilience in challenging conditions, which is relevant for low-gravity environments like Mars.

Despite these promising results, a key limitation is the reduced performance when moving in the supine position and the reliance on a simplified static model. Future research will focus on the practical implementation of advanced adaptive control algorithms, such as the fuzzy logic approach outlined in the discussion, to better manage dynamic variations and ensure more autonomous decision-making. Furthermore, extensive testing on a wider variety of natural terrains is planned to ensure robustness in genuinely unstructured environments.

## Figures and Tables

**Table 1:** The functional role of the articulated bodies in walking robots.

Function	Role of Articulated Body
Balance	Adjusts center of mass for stable locomotion
Efficiency	Stores/reduces energy loads, smooth motion
Maneuverability	Twisting, bending, adapting to terrain
Gait coordination	Syncs leg groups via body movement control
Fault tolerance	Enhances robustness and modularity; Recovery from imbalance or damage
Obstacle navigation	Bends/flexes to go over/under/through barriers

**Table 2:** Hexapod walking robots with multi-segment articulated bodies [16].

Robot	Reference	Design Solution to Achieve Body Flexibility	Supplementary DOF	Layout
AMOS series robots	[7,8]	Single-axis articulation, Rigid connection	γ	2 + 4
Dante II	[15]	Reciprocating mechanism	ψ, X	3 + 3
Hector	[14]	2 DOFs spindle joint	ψ, γ, X	2 + 2 + 2
MELCRAB-2	[17]	Reciprocating mechanism	ψ, X	3 + 3
ModPod	[13]	Multi-axis articulation, Rigid connection	ψ, γ	2 + 2 + 2
ParaWalker	[18]	Stewart platform	Omnidirectional	3 + 3
SpiceClimber	[9]	Single-axis articulation, Rigid connection	γ	2 + 4
Whegs^TM^ series robots	[10,11,12]	Single-axis articulation, Rigid connection	γ	2 + 4

**Table 3:** Actuator-joint kinematic ranges obtained from CAD simulation (front universal joint).

DOF	Actuator Angle Range (deg)	Joint Angle Range—Full (deg)	Monotonic
Pitch (γ)	[−50, +50]	[−53.3, +47.5]	Yes
Roll (ϕ)	[−80, +80]	[−43.1, +56.9]	Yes

Note: The front and rear joints are geometrically identical; the rear mechanism is a mirror image. The same mappings apply in magnitude; the roll sign convention is inverted for the rear joint.

**Table 4:** Joint limits used in the controller (conservative operating range).

DOF	Full Kinematic Range (deg)	Controller Range (deg)	Rationale
Pitch (*γ*)	[−53.3, +47.5]	[−45, +45]	Margin for compliance offsets and end-stop avoidance
Roll (*ϕ*)	[−43.1, +56.9]	[−35, +35]	Margin for compliance offsets and stability

Note: Limits are enforced after summing nominal posture and compliance correction, and before PWM output.

**Table 5:** Control parameters for force-based active compliance outer loop and recovery supervisor (baseline set).

Group	Parameter	Symbol	Value	Unit
Timing	Outer-loop update rate	$f_{c}$	200	Hz
Timing	PWM update rate	$f_{PWM}$	50	Hz
Timing	Sample time	$T_{s}$	0.005	s
Filtering	Force LPF cutoff	$f_{LP}$	8	Hz
Filtering	IIR coefficient	β	0.78	-
Limits	Pitch saturation	$\left[\gamma_{min},\gamma_{max}\right]$	$\left[-45,+45\right]$	deg
Limits	Roll saturation	$\left[\phi_{min},\phi_{max}\right]$	$\left[-35,+35\right]$	deg
Safety shaping	Max. compliance offset (pitch)	${∆\gamma }_{max}$	±10	deg
Safety shaping	Max. compliance offset (roll)	${∆\phi }_{max}$	±8	deg
Safety shaping	Slew-rate limit	${∆\theta }_{max}$	0.5	deg/step
Compliance (stance)	Pitch compliance	$c_{stance}^{\gamma }$	1.003	deg/N
Compliance (stance)	Roll compliance	$c_{stance}^{\phi }$	0.315	deg/N
Compliance (transition)	Pitch compliance	$c_{tr}^{\gamma }$	0.499	deg/N
Compliance (transition)	Roll compliance	$c_{tr}^{\phi }$	0.160	deg/N
Reference	Self-unloading reference	$F^{ref}$	0	N
Supervisor trigger	Pitch overload threshold	$F_{th}^{\gamma }$	12	N
Supervisor trigger	Roll overload threshold	$F_{th}^{\phi }$	25	N
Supervisor trigger	Roll asymmetry threshold	$∆F_{th}^{\phi }$	15	N
Supervisor trigger	Angle-to-limit margin	$\theta_{marg}$	3	deg
Supervisor logic	Enter recovery dwell	$N_{dwell}$	40 (0.20)	samples (s)
Supervisor logic	Exit recovery dwell	$N_{exit}$	60 (0.30)	samples (s)

Note: The force low-pass filter is implemented as a first-order IIR (see Equation (17)); the corresponding coefficient is parameterized as $\beta =e^{-2\pi f_{LP}T_{s}}$. Compliance values follow the design rule $c\approx ∆\theta_{max}/F_{scale}$, where $F_{scale}$ is a characteristic force magnitude observed in experiments. Compliance gains c are reported in deg/N (angles in degrees). Thresholds and dwell times were tuned to avoid chatter.

## Data Availability

The original contributions presented in this study are included in the article. Further inquiries can be directed to the corresponding author.
